# The Role of Antihistamines in the Treatment of Vasomotor Rhinitis

**DOI:** 10.1097/WOX.0b013e3181b35322

**Published:** 2009-08-15

**Authors:** Phil Lieberman

**Affiliations:** 1 Department of Medicine and Pediatrics, University of Tennessee, Germantown, TN

**Keywords:** antihistamines, vasomotor rhinitis, nonallergic rhinitis, anti-inflammatory activities, nonallergic activities

## Abstract

**Background:**

The pathogenesis of vasomotor rhinitis is not understood. It is unlikely that antihistamines, based on their H1 antagonist activity alone, would be effective in this disorder.

**Methods:**

Nonetheless, at least one double-blind, placebo-controlled multicenter trial has found that intranasal azelastine relieves symptoms of this disorder better than placebo. The mechanism responsible for its beneficial effect in nonallergic rhinitis is unclear but probably relates to "anti-inflammatory/antiallergic" activities.

**Results:**

Such mechanisms have been demonstrated for a number of different oral antihistamines, but often the concentrations required in vitro are higher than those that are normally achieved in vivo using recommended dosing. It has been postulated that intranasal administration, which can achieve high local levels, might be a factor responsible for enhancing the "anti-inflammatory/antiallergic" properties.

**Conclusions:**

Interpreting this information allows one to conclude that antihistamines may be potentially effective agents in vasomotor rhinitis, and are more likely to be so when administered intranasally, despite the fact that data documenting this beneficial effect are sparse.

## Introduction

Perhaps the best way to introduce the topic of the role of antihistamines in the management of vasomotor rhinitis is to quote a statement from the ARIA Workshop Reports [1]. In a section entitled, "Noninfectious, Nonallergic Rhinitis," the ARIA Report states: "Our knowledge in this area is poor. The response to H1-antihistamines in patients who have sneezing as a predominant symptom points at histamine as an important mediator, but H1-antihistamines are generally ineffective in most patients." Why then a review of the role of antihistamines in the management of vasomotor or nonallergic rhinitis?

The reason for the inclusion of antihistamines as a potentially beneficial therapeutic agent in vasomotor rhinitis (VMR) is a seminal study by Banov et al [2] demonstrating that azelastine was effective in managing this disorder. The Banov article was published in the same year as the ARIA guidelines (2001), and, thus, was not included in the ARIA document. This article changed our perspective on the potential use of antihistamines, at least those administered intranasally, in the treatment of VMR [3-6]. In addition, a suggestion that oral antihistamines might be helpful in the treatment of VMR was generated by Purello-D'Ambrosio et al [7]. The Purello-D'Ambrosio study compared loratadine + flunisolide to flunisolide + placebo in patients suffering from a subcategory of nonallergic rhinitis (NAR), nonallergic rhinitis with eosinophilia (NARES). The loratadine + flunisolide treated group fared better than the flunisolide + placebo group. Improvements were noted in sneezing and rhinorrhea, prompting the authors to conclude that loratadine "improves the effectiveness of flunisolide in treatment of NARES." Thus, there clearly is justification for a review of antihistamine therapy in the management of VMR.

## Some Putative Mechanisms of Action of Antihistamines in the Treatment of VMR

The proposed mechanisms by which antihistamines might be effective in the therapy for VMR are include:

1. "Classic" H1 antagonist (reverse agonist) activity as believed to be responsible for their beneficial effects in allergic rhinitis

2. Antagonism of other histamine receptors, most probably via the H2 and, perhaps, H3 receptors

3. Other "anti-inflammatory/antiallergic" actions

A discussion of each of these potential mechanisms of action can shed some light on the observations, noted above,[2,7] that antihistamine treatment may have a salutary effect on the symptoms of VMR.

### Potential Benefit via H1 Receptor Antagonism

Histamine has 4 receptor types (H1, H2, H3, and H4), which are all G protein-coupled [8]. Antihistamines used in the treatment of allergic rhinitis all have as their most prominent activity, antagonism (reverse agonism) of the H1 receptor. The characteristics of the H1 receptor are shown in Table 1. The difficulty in postulating a role for H1 antagonism in the treatment of nonallergic rhinitis is the scant evidence that histamine plays a role in this disorder [9]. Thus, it is unlikely that the demonstrated effects of antihistamines on VMR symptoms are related to H1 receptor antagonism (reverse agonism).

**Table 1:** Histamine and Its Activities

Receptor	H_1_
Signal conduction through	G_q/11 _and others
Location of receptors	Multiple sites throughout the body including: Smooth muscle bronchi and gastrointestinal tract, cardiac tissue, blood vessels, sensory nerves, endothelium, central nervous system
Chromosome location	3p25, 3p14-21
Signal conduction induces	Increased cyclic GMP, increased intracellular cytosolic calcium, activation of phospholipase C, activation of guanyl cyclase, nitric oxide production
Antagonists (reverse agonists)	Over 40 exist. Examples of "second generation" include cetirizine, desloratadine, fexofenadine, loratadine, azelastine, olopatadine Examples of "first generation" antihistamines are chlorpheniramine, diphenhydramine, pyribenzamine, and others
Activities	Increases vascular permeability producing a fall in blood pressure, flush, headache, and reflex tachycardia; itch; smooth muscle contraction in bronchi and gastrointestinal tract; stimulation of vagal nerve receptors producing reflex smooth muscle contraction in airways; cough via stimulation of sensory nerves in airways; eosinophil chemotaxis; decreased AV node conduction time; enhancement of release of histamine and arachidonic acid derivatives; nitric oxide formation
Nasal symptoms produced	Sneezing, itching, rhinorrhea, and perhaps some degree of nasal congestion via increased vascular permeability with leakage of fluid into the tissues and vasodilatation?

### Antagonism of the Effects of H2, H3, and H4 Receptors

#### The H2 Receptor

The H2 receptor is a member of the heptahelical receptor family (Table 2). There is some evidence that H2 receptors mediate dilation of nasal blood vessels [10]. Wood-Baker et al showed that stimulation of the H2 receptor can cause nasal obstruction presumably because of vasodilatation. Subjects were pretreated with oral cetirizine or ranitidine in a double-blind, randomized manner after which histamine was applied to the nasal mucosa. They measured the concentration of total protein and albumin in nasal lavage fluid and nasal airway resistance before and after histamine challenge. Ranitidine reduced the maximum increase in nasal airway resistance, but this effect was only significant when combined with cetirizine. The increase in nasal lavage protein and albumin was almost completely abolished by cetirizine; ranitidine had far less effect. They concluded that H2 receptor antagonism had little, if any, effect on vascular permeability but did seem to have some effect on nasal obstruction.

**Table 2:** H2 Receptor Antagonists

Receptor	H_2_
Signal conduction through	G_s_
Location of receptors	Widely expressed including: Mucosa of stomach, cardiac tissue, uterus, smooth muscle vascular bed, epithelium of mucosa of nose, submucosal glands in nose, central nervous system, immune cells
Chromosome location	5q35.3
Signal conduction induces	Increase in cyclic AMP, activation of adenyl cyclase
Antagonists (reverse agonists)	Burimamide, cimetidine, dimaprit, famotidine, nizatidine, ranitidine, and others (it should be noted that a number of different H1 antagonists also show affinity for the H2 receptor
Activities	Increased gastric acid secretion; increases vascular permeability producing a fall in blood pressure, flush, headache, and reflex tachycardia; stimulate mucus production in the lungs; direct chronotropic effect on atrium and inotropic action on ventricle; relaxation of esophageal sphincter; stimulation of suppressor T-cells; decrease in neutrophil and basophil chemotaxis and activation; proliferation of lymphocytes; activity of NK cells
Nasal symptoms produced	Antagonism of the H2 receptor could potentially reduce the effect of histamine on nasal airway swelling, producing nasal decongestion

Further evidence was found for a role of H2 receptor stimulation in the production of symptoms of rhinitis via an investigation by Shelton and Eisor [11]. These investigators applied histamine, a specific H1 receptor agonist (betahistine, and a specific H2 receptor agonist) impromidine, to the nasal mucosa of 11 normal subjects and 4 patients with rhinitis. Sneezing, nasal irritation, and hypersecretion were induced only by histamine and the H1 receptor agonist, betahistine. Nasal airway resistance was increased by all 3 agents, with histamine being the most potent effector and with the H2 receptor agonist, impromidine, producing a greater effect than the H1 receptor agonist, betahistine. Thus, this study clearly showed a role for H2 receptor stimulation in the production of nasal congestion.

A number of H1 antagonists demonstrate H2 receptor binding. For example, Sharif et al compared the activity of several H1 antagonists on the H2 receptor [12]. They evaluated pyrilamine, ketotifen, pheniramine, antazoline, olopatadine, and levocabastine for their affinities for the H1, H2, and H3 receptors and found promiscuity among the drugs, demonstrating that H1 receptor antagonists can also bind H2 and H3 receptors.

Bielory et al [13] reviewed the relative H1 and H2 receptor affinities of several H1 antagonists and reported a hierarchy of affinities for each receptor (Table 3).

**Table 3:** Relative Histamine Receptor Binding Affinities (Ki) for Selected H1 Antagonists

H1 Antagonist	H1 Receptor (nM)	H2 Receptor (nM)	H3 Receptor (nM)
Ketotifen	1.3	1155	2277
Emedastine	1.3	49,067	12,430
Desloratadine	4	ND	ND
Cetirizine	6.3	ND	ND
Azelastine	6.8	ND	ND
Epinastine	9.8*	4030*	ND
Diphenhydramine	12.5	1600	25,000
Olopatadine	32	100,000	79,400
Loratadine	35	ND	ND
Levocabastine	56	23,500	4597
Fexofenadine	83	ND	ND
Pyrilamine	0.8	9510	1016
Chlorpheniramine	1.4	7980	3103
Pheniramine	34	14,567	10,567
Antazoline	38	44,433	42,400
Ranitidine	46,100	187	10,537
Cimetidine	6190	2377	20,750
Thioperamide	280,000	57,967	1.1
Methylhistamine	138,000	72,100	1.4
Histamine	180,000	18,350	4.5

Ki is the dissociation constant based on the Cheng-Prussof equation, Ki 1/4 IC50/(1 Þ L/Kd), such that a lower value denotes higher affinity binding. IC50 is the concentration of drug needed to produce 50% inhibition of the receptor binding.

The lower the value the higher the affinity (13).

ND, no data.

*Reported as the IC_50_.

Thus, at least on a theoretical basis, antagonism of the H2 receptor could have some effect on the nasal congestion of VMR; and H1 antagonists can exert antagonism on the H2 receptor. Once again, however, a word of caution: there is no clear-cut evidence that histamine plays a role in producing symptoms of VMR.

#### The H3 Receptor

The H3 receptor is a G protein-coupled receptor (Table 4) located primarily at presynaptic sites on histaminergic nerve terminals. Histamine stimulates these terminals causing inhibition of activity. In the nose, by acting on the H3 receptor, histamine can reduce the release of norepinephrine from sympathetic nerves, an effect that could diminish the tonic role of sympathetic stimulation in the homeostatic maintenance of nasal airway patency.

**Table 4:** H3 Receptor Antagonists

Receptor	H_3_
Signal conduction through	G_i/o_
Location of receptors	Mainly expressed on presynaptic nerves in the peripheral sympathetic adrenergic system and histaminergic nerves in the central nervous system. Receptors can be found in airways and gastrointestinal tract
Chromosome location	20q13.33
Signal conduction induces	Decreased cyclic AMP, induction MAP kinase
Antagonists (reverse agonists)	None available clinically: Thioperamide, alobenpropit, and others Very little data regarding effect of H1 antagonists on the H3 receptor are available (13)
Activities	Opposes bronchoconstriction and gastric acid; suppression of norepinephrine release at presynaptic nerve endings
Nasal symptoms produced	Can produce nasal decongestion by blocking effect of histamine on adrenergic postsynaptic receptors

H3 antagonists have been shown to reverse nasal congestion induced by the intranasal application of histamine. Taylor-Clark et al [14] investigated the mechanisms by which histamine produced nasal blockage. They applied histamine and specific histamine receptor agonists intranasally in healthy human subjects and assessed their effect on airway congestion by acoustic rhinometry. Oral pretreatment with cetirizine had only a partial effect on nasal blockage. Dimaprit, an H2 agonist, produced nasal congestion that was reversed by ranitidine. A combination of cetirizine and ranitidine caused greater inhibition of nasal blockage than cetirizine alone. An H3 agonist, R-alpha-methylhistamine, produced nasal blockage that was not inhibited by cetirizine or ranitidine.

The H3 antagonist, thioperamide, reversed the R-alpha-methylhistamine induced nasal blockage. Thioperamide alone had no effect on nasal blockage caused by histamine, but in the presence of cetirizine, thioperamide further reduced the histamine-induced nasal blockage. They concluded that nasal congestion because of the application of histamine to healthy nasal mucosa results from activity at H1, H2, and H3 receptors.

Therefore, there is a third potential means by which antagonism of histamine could favorably affect nasal congestion. However, again, for this therapy to be effective, histamine would have to play a role in producing congestion in VMR, and H1 antagonists would have to show some promiscuity, being active at the H2 and H3 receptors as well.

#### The H4 Receptor

The H4 receptor (Table 5) is mentioned only for the sake of thoroughness. H4 receptors can in many instances be considered anti-inflammatory. They are found on a number of cells, and down-regulate histamine-induced inflammatory responses in these cells. There is no evidence that H1 antagonists act at the H4 receptor. Thus, little can be said at this time about the potential role of H4 antagonism in the therapy for VMR.

**Table 5:** H4 Receptor Antagonists

Receptor	H_4_
Signal conduction through	G_i/o _and G_15/16_
Location of receptors	Eosinophils, neutrophils, basophils, mast cells, spleen, liver, lung colon, epicanthus, and bone marrow
Chromosome location	18q11.2
Signal conduction induces	Decreased cyclic AMP, MAP kinase induction, phospholipase C activation, formation of diacylglycerol, calcium mobilization
Antagonists (reverse agonists)	None available clinically: Has homology with H_3 _receptor and is also antagonized by same drugs
Activities	Chemotaxis and chemokinesis of mast cells and eosinophils; enhancement of the activity of other chemoattractants (eg, chemokines) on eosinophils; upregulation of adhesion molecules
Nasal symptoms produced	Since histamine can up regulate inflammation on cells such as eosinophils, H4 antagonists may have a beneficial role in this regard

### Anti-Inflammatory Activities of Antihistamines

It is well known that antihistamines have effects which have been called "anti-inflammatory" or "antiallergic." These effects have been well documented and have been demonstrated for many different molecules including those administered orally and by intranasal application. Some of the in vitro effects may be of very little clinical importance because, in many instances, the concentration of antihistamine necessary to produce the effects are higher than those commonly achieved by oral administration of recommended doses. However, in some instances, for intranasally administered antihistamine, the concentrations achieving an "anti-inflammatory effect" are obtained at recommended doses. This may explain the fact that the only antihistamine to date approved for use in VMR in the US is azelastine, which is administered intranasally. Some of the presumably most important "anti-inflammatory/antiallergic" activities of antihistamines are noted in Table 6 and summarized below.

**Table 6:** "Anti-inflammatory/Antiallergic" Activities of Antihistamines

Prevent mast cell degranulation
Downregulate expression of adhesion molecules
Downregulate chemotaxis
Eosinophils
Neutrophils
Enhance inflammatory cell apoptosis
Reduce inflammatory cytokine expression
Decrease neurogenic enhancement of inflammation

### Prevention of Mast Cell/Basophil Degranulation

Perhaps the most well studied anti-inflammatory/anti-allergic effect of antihistamines is related to their ability to inhibit mast cell and basophil mediator release. This effect has been demonstrated both in vitro and in vivo [8]. Both preformed mediators and newly synthesized mediator release can be prevented by antihistamines. This effect has been shown not only for IgE-mediated release, induced by both allergen and anti-IgE, but also for secretagogues including compound 48/80, substance P, concanavalin A, and calcium ionophore A23187.

In general, all second-generation antihistamines show some inhibition of inflammatory mediator release from mast cells and basophils in vitro, but there is a great deal of heterogeneity between activity of these drugs in terms of the concentration required, the experimental conditions employed, and the degranulation stimulus.

For example, Okayama et al studied the effects of terfenadine, ketotifen, and cetirizine on human lung, tonsil, and skin mast cells stimulated immunologically with anti-IgE, and found varying results depending on the drug studied [15].

Both azelastine and olopatadine have clearly been shown to diminish basophil and mast cell degranulation. Azelastine has been shown to inhibit the release of histamine from both human and animal mast cells induced by various stimuli including anti-IgE, antigen, calcium ionophores, and compound 48/80. In vivo this agent has been demonstrated to prevent synthesis and/or release of mediators including leukotrienes, tosyl arginine, methyl esterase, oxygen free radicals, platelet activating factor, and superoxide anion [16].

Olopatadine has also shown mast cell stabilizing activity inhibiting the release of histamine, tryptase, tumor necrosis factor-*α*, and prostaglandin D2 [17]. Thus, the prevention of mast cell and basophil degranulation is a well documented characteristic of a number of different antihistamines. With intranasal antihistamines, this effect has been shown to occur in vivo after intranasal application in human beings.

### Down-Regulation of the Expression of Adhesion Molecules

The down-regulation of intracellular adhesion molecule ICAM-1 has been demonstrated for levocabastine, fexofenadine, terfenadine, and desloratadine in vitro [18]. Perhaps more important, because of the greater clinical applicability, is the ability of antihistamines to reduce the expression of ICAM-1 and the subsequent inflammatory cell infiltration in the eyes and nose in vivo. Azelastine, levocabastine, cetirizine, oxatomide, terfenadine, fexofenadine, mizolastine, olopatadine, and loratadine have all been shown to diminish ICAM-1 expression on either nasal and/or conjunctival epithelial cells. This can result in a reduction of inflammatory infiltrate into these mucosal tissues. These effects have been observed after both allergen challenge and during natural antigen exposure [18].

### Regulation of Chemotaxis

Fexofenadine, ketotifen, cetirizine, loratadine, terfenadine, and desloratadine have been shown to diminish eosinophil chemotaxis in vitro, and ketotifen, terfenadine, azelastine, and cetirizine have demonstrated a decrease in neutrophil chemotaxis in vivo [8].

### Enhancement of Apoptosis of Inflammatory Cells

Enhancement of programmed cell death for eosinophils has been demonstrated for ketotifen and cetirizine [8]. In addition, azelastine administered orally has been found to reduce peripheral eosinophilia in a patient with the hypereosinophilic syndrome. Ito, et al, reported a case of hypereosinophilic syndrome in a 9-year-old, in which the administration of azelastine significantly reduced peripheral eosinophilia while simultaneously markedly diminishing the serum interleukin 5 levels [19].

### Reduction of the Inflammatory Cytokine Expression

As with the other anti-inflammatory/antiallergic effects noted above, antihistamines have been shown to diminish the expression of a number of different cytokines, both in vivo and in vitro. These effects have been shown for IL4, IL6, IL8, IL13, and tumor necrosis factor-*α *and other inflammatory cytokines [8,20].

### Suppression of Neurogenic Enhancement of Inflammation

Neurogenic inflammation or an imbalance in neural control of the nasal airways has been postulated to play a role in the production of VMR [9]. Inhibition of the inflammatory effects of neurogenic stimulation therefore might be expected to diminish symptoms of VMR. Antihistamines have been shown to favorably affect neurogenic inflammation. For example, substance P has been shown to be released during antidromic stimulation. Oral administration of azelastine has been found to reduce substance P levels in bronchial lavage and nasal washings produced by allergen challenge [21].

Thus, in summary, antihistamines have been shown to exert a number of different anti-inflammatory/antiallergic effects. These effects could clearly have a salutary effect on nasal symptoms. It has been shown that at least some patients with VMR demonstrate inflammatory infiltrates in the nasal mucosa [22-25]. Therefore, it would not be unreasonable to assume that inhibition of the inflammatory activity resulting in these infiltrates could be responsible for the improvement in VMR symptoms shown in at least one double-blind placebo-controlled study [2].

## Clinical Implications

Based on a summation of the above information, there is little doubt that antihistamine therapy could be potentially effective in the treatment of VMR, especially through anti-inflammatory/antiallergic effects. However, there has been a dearth of clinical confirmation of the efficacy of antihistamines, especially oral antihistamines, in VMR.

### Oral Antihistamines

Trials of oral antihistamines are scarce and have yielded, in general, inconsistent and for the most part, disappointing results [7,26-28]. Two of these studies evaluated an antihistamine as adjunctive therapy either with a decongestant [26] or a topical steroid [7]. Broms and Malm found that phenylpropanolamine alone in a dose of 100 mg reduced nasal airway resistance, but that this drug in a dose of 50 mg combined with an oral antihistamine had no effect on nasal airway resistance [26]. Purello-D'Ambrosio [7] found loratadine added to the beneficial effect of flunisolide. Two other studies looked at antihistamines as monotherapy [27,28]. Mullarkey [27] reported that topical steroids were superior to antihistamines in the treatment of patients with NARES; Rinne et al [28] came to a similar conclusion in a long-term study comparing topical budesonide to cetirizine in NARES patients.

### Intranasal Antihistamines

The only antihistamine currently approved in the US for use in VMR is azelastine, an agent with broad-based pharmacologic activities in addition to its H1 receptor antagonist action. Food and Drug Association (FDA) approval was based on 2 pivotal double-blind, placebo-controlled trials demonstrating efficacy [2]. Another multicenter, double blind, randomized, parallel-group study by Gehanno et al confirmed the efficacy of azelastine in VMR,[22] and 2 large open-label studies employing patient questionnaires demonstrated the beneficial effect of azelastine in patients with allergic rhinitis, mixed rhinitis, and VMR [23,24].

There is also a study employing topical levacobastine in a small number of patients, some with allergic rhinitis and some with VMR. Levocabastine was found to be superior to placebo for symptoms of nasal discharge and sneezing but not for congestion in the VMR group [25].

### A Closer Look

Because the FDA approval of azelastine for the treatment of nonallergic rhinitis was granted based on 2 studies, as noted above, by Banov, et al, [2] it would be of value to take a closer look at these investigations. Both studies were multicenter placebo-controlled trials of azelastine for the treatment of nonallergic VMR, and both used identical protocols. More than 200 patients were evaluated; the response rates were between 82 and 85%. It is of note that the response to placebo in these studies was quite high, 73%. This is not unexpected because placebo saline nasal spray has shown to exert some beneficial effect in this disorder.

All of the patients in these trials had experienced symptoms of VMR for at least one year, had negative skin tests to a mixed panel of seasonal and perennial allergens, and had no eosinophils as evaluated by nasal cytology. After a 1 week, single-blind, placebo lead-in period, patients who met the symptom severity qualification criteria were randomized to receive either azelastine nasal spray or placebo. Patients recorded the severity of their symptoms on diary cards each morning and evening, using a 4-point symptom rating scale. The primary efficacy variable was the overall reduction from baseline in the total VMR symptom score over the 21 day, double-blind, treatment period.

In both trials, azelastine was superior to placebo in reducing VMR symptom score from baseline (study 1, *P *= 0.002; study 2, *P *= 0.005). Significant improvement occurred within the first week. In addition, of note is that azelastine was superior to placebo in all 4 symptoms measured, including nasal congestion.

## Conclusions

Data are sparse regarding the effects of antihistamines on the symptoms of VMR. Nonetheless, there is theoretical rationale, based upon the activities of antihistamines (especially their anti-inflammatory/antiallergic activities) to support the hypothesis that they may be effective agents in treating VMR. This hypothesis was confirmed in a study of azelastine in the treatment of this disorder. However, to date there is no convincing evidence that oral antihistamines are helpful, in general, as monotherapy and only inconsistent and sparse data supporting their use as adjunctive treatment given with topical coticosteroids or decongestants.

## End Note

Dr. Lieberman discloses that he is a consultant, or on an advisory board or the speakers bureau for GlaxoSmithKline (GSK), Sanofi-Aventis Pharmaceuticals, Schering Plough Corporation, Meda Pharmaceuticals, and Alcon Laboratories.

Presented at a roundtable conference held in December 2008 in Washington, DC. The meeting was sponsored by the TREAT Foundation (Washington, DC) and supported through an unrestricted educational grant from Meda Pharmaceuticals. The funding company did not have any input into the development of the meeting or the series, and the company was not represented at the roundtable meeting.
